# Engagement in Advance Care Planning: The Association With Cognitive Impairment and Care Preferences

**DOI:** 10.1093/geroni/igaa057.1347

**Published:** 2020-12-16

**Authors:** Hyo Jung Lee, Giyeon Kim

**Affiliations:** 1 Nanyang Technological University, Singapore, Singapore; 2 Chung-Ang University, Seoul, Republic of Korea

## Abstract

Although there has been growing evidence that Advance care planning (ACP) benefits people with cognitive impairment nearing death, our understanding about this issue is still limited. This study examines whether cognitive impairment is associated with ACP engagement and end-of-life care preferences among older adults in the U.S. Using data from the 2012 National Health and Trends Study (n=1798, aged 65 to 101), we identified four levels of ACP engagement: None (28%), Informal ACP conversation only (12%), Formal ACP only (14%), and Both informal and formal ACP (46%). Older adults with None showed the highest prevalence of having cognitive impairment (17%), followed by those with Formal ACP only (15%) and the other two (6%, 6%). The results of Multinomial Logistic Regression showed that, compared to those without, respondents with cognitive impairment had 143% increased relative risk of having None (RR = 2.43, CI: 1.58-3.73) and 81% increased relative risk of completing Formal ACP only (RR = 1.81, CI: 1.11-2.95) relative to completing Both informal and formal ACP. In addition, respondents with None were more likely to prefer to receive all treatments available nearing death than those with any ACP engagement. Achieving high quality care at the end of life can be more challenging for older adults with cognitive impairment and their family caregivers due to the limited capacity. Although encouraged, informal ACP conversation with loved ones does not necessarily occur before the formal ACP, especially, for those with cognitive impairment. Therefore, they may merit more attention such as early ACP engagement.

